# Effectiveness Associated With Vaccination After COVID-19 Recovery in Preventing Reinfection

**DOI:** 10.1001/jamanetworkopen.2022.23917

**Published:** 2022-07-27

**Authors:** Nickolas Lewis, Laura C. Chambers, Huong T. Chu, Taylor Fortnam, Roberta De Vito, Lisa M. Gargano, Philip A. Chan, James McDonald, Joseph W. Hogan

**Affiliations:** 1 Brown University School of Public Health, Providence, Rhode Island; 2 Rhode Island Department of Health, Providence; 3 Brown University Department of Medicine, Providence, Rhode Island

## Abstract

**Importance:**

The benefit of vaccination for preventing reinfection among individuals who have been previously infected with SARS-CoV-2 is largely unknown.

**Objective:**

To obtain population-based estimates of the probability of SARS-CoV-2 reinfection and the effectiveness associated with vaccination after recovery from COVID-19.

**Design, Setting, and Participants:**

This cohort study used Rhode Island statewide surveillance data from March 1, 2020, to December 9, 2021, on COVID-19 vaccinations, laboratory-confirmed cases, hospitalizations, and fatalities to conduct a population-based, retrospective study during periods when wild type, Alpha, and Delta strains of SARS-CoV-2 were predominant. Participants included Rhode Island residents aged 12 years and older who were previously diagnosed with COVID-19 and unvaccinated at the time of first infection, stratified into 3 subpopulations: long-term congregate care (LTCC) residents, LTCC employees, and the general population (ie, individuals not associated with congregate settings). Data were analyzed from October 2021 to January 2022.

**Exposures:**

Completion of the primary vaccination series, defined as 14 days after the second dose of an mRNA vaccine or 1 dose of vector virus vaccine.

**Main Outcomes and Measures:**

The main outcome was SARS-CoV-2 reinfection, defined as a laboratory-confirmed positive result on a polymerase chain reaction (PCR) or antigen test at least 90 days after the first laboratory-confirmed positive result on a PCR or antigen test.

**Results:**

Overall, 3124 LTCC residents (median [IQR] age, 81 [71-89]; 1675 [53.6%] females), 2877 LTCC employees (median [IQR] age, 41 [30-53]; 2186 [76.0%] females), and 94 516 members of the general population (median [IQR] age, 35 [24-52] years; 45 030 [47.6%] females) met eligibility criteria. Probability of reinfection at 9 months for those who remained unvaccinated after recovery from prior COVID-19 was 13.0% (95% CI, 12.0%-14.0%) among LTCC residents, 10.0% (95% CI, 8.8%-11.5%) among LTCC employees, and 1.9% (95% CI, 1.8%-2.0%) among the general population. Completion of the primary vaccination series after infection was associated with 49% (95% CI, 27%-65%) protection among LTCC residents, 47% (95% CI, 19%-65%) protection among LTCC employees, and 62% (95% CI, 56%-68%) protection in the general population against reinfection, adjusting for potential sociodemographic and clinical confounders and temporal variation in infection rates.

**Conclusions and Relevance:**

These findings suggest that risk of SARS-CoV-2 reinfection after recovery from COVID-19 was relatively high among individuals who remained unvaccinated. Vaccination after recovery from COVID-19 was associated with reducing risk of reinfection by approximately half.

## Key Points

**Question:**

How effective is vaccination against COVID-19 after recovery from prior SARS-CoV-2 infection?

**Findings:**

In this cohort study of more than 95 000 Rhode Island residents from March 2020 to December 2021, including residents and employees of long-term congregate care (LTCC) facilities, completion of the primary vaccination series after recovery from COVID-19 was associated with 49% protection from reinfection among LTCC residents, 47% protection among LTCC employees, and 62% protection in the general population during periods when wild type, Alpha, and Delta strains of SARS-CoV-2 were predominant.

**Meaning:**

These findings suggest that among people who have recovered from COVID-19, subsequent completion of the primary vaccination series reduced the risk of reinfection by approximately half.

## Teaser

This cohort study assesses the association of vaccination with protection against SARS-CoV-2 reinfection among long-term congregate care residents and employees and the general population previously infected with SARS-CoV-2 in Rhode Island.

## Introduction

Current evidence suggests that individuals who experience a SARS-CoV-2 infection are susceptible to reinfection.^1,2^ While evidence suggests that rates of hospitalization and death are substantially lower in individuals who have been reinfected,^1^ the dynamics of SARS-CoV-2 reinfection are still poorly understood. A prior infection of SARS-CoV-2 confers some immunity to individuals who are not vaccinated,^3,4^ but this natural immunity wanes over time. Additionally, it has been shown that the JNJ-78436735 (Janssen), mRNA-1273 (Moderna), and BNT162b2 (Pfizer-BioNTech) vaccines offer substantial protection against COVID-19 infection and remain effective against newer variants.^5,6,7^

A prior case-control study of Kentucky residents found that individuals who had been previously infected with completion of the primary vaccination series (CPVS) were better protected against reinfection than individuals who remained unvaccinated.^8^ Similar results have been reported in larger cohort studies in Israel and United Kingdom.^9,10^ Our primary goal was to quantify the effectiveness associated with vaccination against reinfection among individuals who had been previously infected with SARS-CoV-2 prior to vaccination. Secondary goals were to estimate the probability of reinfection at 3, 6, and 9 months after recovery from primary infection and to assess the associations of measurable risk factors with reinfection. Our analysis has particular relevance to the ongoing discussion about vaccination requirements for individuals who have recovered from COVID-19.

## Methods

This cohort study was classified as exempt from review and informed consent by the Rhode Island Department of Health (RIDOH) institutional review board because we used existing data that were deidentified. This study follows the Strengthening the Reporting of Observational Studies in Epidemiology (STROBE) reporting guideline for observational studies.

### Study Design and Population

**Figure 1.**
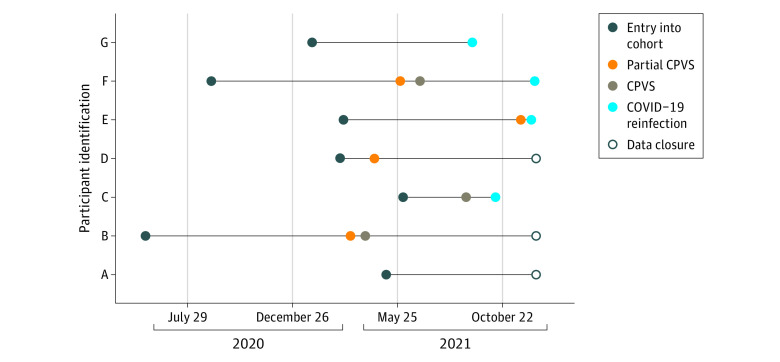
Sample of Observation Patterns CPVS indicates completion of primary vaccination series.

We conducted a retrospective population-based cohort study using statewide surveillance data collected by the RIDOH between March 1, 2020, and December 9, 2021. The study population included Rhode Island residents aged 12 years and older who were unvaccinated at the time of their first laboratory-confirmed SARS-CoV-2 infection and who remained unvaccinated at least 90 days after the initial positive test result date. We excluded people younger than age 12 years because they did not become eligible for vaccination until the final month of the analysis period. Example patient timelines are detailed in Figure 1.

**Figure 2.**
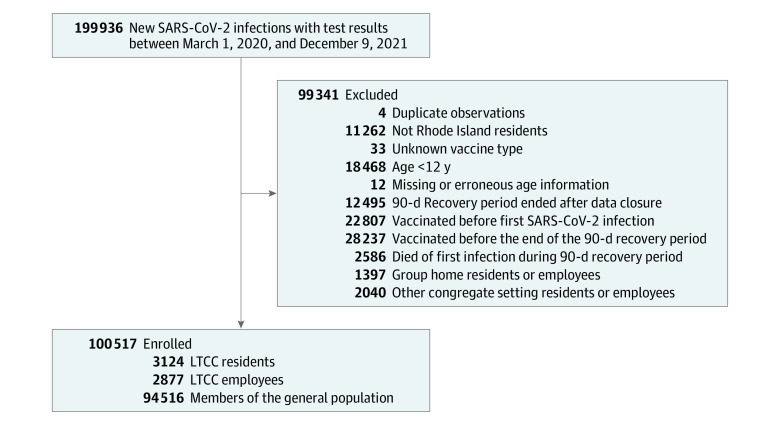
Flowchart for the Analysis Cohort LTCC indicates long-term congregate care.

The initial data set included 199 936 individuals. We excluded 11 262 non–Rhode Island residents, 18 468 individuals who were younger than age 12 years, 12 individuals who had missing or erroneous age information, and 51 044 individuals who were either vaccinated prior to their first laboratory-confirmed infection or before the 90-day recovery period for their first infection had ended (Figure 2).

We divided our sample into 3 separate subpopulations: (1) residents of long-term congregate care (LTCC) settings, which included nursing homes and assisted living facilities, (2) employees of LTCC settings, and (3) the general population (ie, individuals not associated with congregate settings). LTCC residents were analyzed separately owing to the high risk of morbidity and mortality in this population, and LTCC employees were analyzed separately because they worked for extended periods of time in these high-transmission environments. We did not include group home residents or employees (2040 individuals), other congregate setting residents or employees (1397 individuals), and individuals with unknown congregate status in this study. Other congregate settings included predominantly correctional facilities. After exclusions, our analysis sample included 3124 LTCC residents, 2877 LTCC employees, and 94 516 members of the general population.

### Data Management

The study database was assembled by linking data from 4 surveillance systems maintained by RIDOH: COVID-19 vaccinations, cases, hospitalizations, and fatalities. COVID-19 vaccination doses administered were identified based on the Rhode Island Child and Adult Immunization Registry (RICAIR) data system. In Rhode Island, positive polymerase chain reaction (PCR) and antigen (prior to widespread availability of home-based rapid antigen tests) tests are reported to RIDOH, and new cases are investigated to collect health and demographic information. Using RIDOH’s statewide Hospital Incident Reporting System, hospitals report to RIDOH patients who are admitted to an inpatient bed and are being placed on COVID-19 precautions. RIDOH derives data for laboratory-confirmed COVID-19-associated fatalities from 3 sources: the Office of the State Medical Examiners, vital records death certificate data, and the Hospital Incident Reporting System.

### Statistical Analysis

We conducted a survival analysis to characterize the risk of reinfection by subpopulation and to estimate the effectiveness associated with vaccination in preventing reinfections among people who have recovered from prior COVID-19. We assumed that it takes 90 days to recover from a previous infection,^11^ starting from the date of the positive test results for the first infection. After the 90-day recovery period, individuals who have remained unvaccinated were considered eligible to enter the risk cohort for COVID-19 reinfection. Individuals were then followed from entry time to time of second infection. Individuals who died after entry or did not experience a second infection by December 9, 2021, were considered censored (Figure 1). Vaccination status was recorded each day and categorized as (1) CPVS (2 doses of mRNA-1273 or BNT162b2 vaccine or a single dose of JNJ-78436735 vaccine), (2) partially CPVS (single dose of mRNA-1273 or BNT162b2), or (3) unvaccinated. Individuals were classified as CPVS or partially CPVS 14 days after administration of any dosage of a vaccine.^12^

For each subpopulation, we calculated the 3-, 6-, and 9-month probability of reinfection by vaccination status using the Kaplan-Meier method. We also calculated incidence rates by dividing the number of reinfections during follow-up by the number of person-days spent in each vaccination category. For this calculation, a person with CPVS with either mRNA-1273 or BNT162b2 contributed person-time to all 3 categories (Figure 1).

We used the Cox proportional hazards regression model to estimate vaccine effectiveness, adjusting for age (years), sex assigned at birth (female, male, unknown), race and ethnicity (self-reported as Asian, Black, Hispanic or Latino, White, and other [including American Indian or Alaskan Native, Native Hawaiian or Other Pacific Islander, other race, or multiple races] or unknown race), previous hospitalization during the first infection, symptom status during the first infection (ie, asymptomatic, symptomatic, or unknown), and time of entry into the risk cohort for reinfection relative to the beginning of the pandemic in Rhode Island (in weeks). Race and ethnicity were included because of previously documented disparities in infection risk.^13^ We also included a zip code–based 3-tier community risk classification created by RIDOH to help guide COVID-19 surveillance and response efforts (low-, moderate-, and high-risk tiers). The tier classification is based on community characteristics, such as population density, sociodemographic characteristics, and COVID-19 burden. Within each model, the interaction of age and time of entry into the risk cohort for reinfection were captured using cubic splines with *df* = 4. Cubic splines were used to capture nonlinear associations. Of note, case investigations for residents of LTCC settings generally occurred through a single point of contact at each facility, rather than individual discussions with residents who were diagnosed with COVID-19, leading to heterogeneity in the level of detail on sociodemographic and clinical characteristics. For each of the 3 subpopulations, we estimated vaccine effectiveness (VE) associated with preventing COVID-19 reinfection as *VE* = 1 – *AHR*,^7,14,15^ where *AHR* is the adjusted hazard ratio estimated by the proportional hazards model.

Our sample included all cases from March 2020 to December 2021, meaning that the VE estimates partially depend on data from a period during which vaccination was not yet available. To assess the robustness of our findings to sample selection, we conducted a supplementary sensitivity analysis in which we restricted to individuals whose first infection occurred during or after December 2020, when vaccines first became available.

Statistical analyses were conducted with R statistical software version 4.1.2 (R Project for Statistical Computing) using the ‘survival’ package for Kaplan-Meier and Cox proportional hazards model. Data were analyzed from October 2021 to January 2022.

## Results

Between March 1, 2020, and December 9, 2021, 100 517 Rhode Island residents aged 12 years and older had recovered from SARS-COV-2 infection and remained unvaccinated at the end of their 90-day recovery period. During the subsequent study follow-up period, a total of 56 888 people (56.6%) received at least 1 dose of a vaccine (4742 individuals received JNJ-78436735, 34 883 individuals received BNT162b2, and 17 263 individuals received mRNA-1273), including 2077 of 3124 LTCC residents (66.4%), 2133 of 2877 LTCC employees (74.1%), and 52 687 of 94 516 members of the general population (55.7%). Sociodemographic and clinical characteristics of the subpopulations are shown in Table 1. The sample of LTCC residents had a median (IQR) age 81 (71-89) years. A substantial percentage of LTCC residents were missing data on symptom status during the first infection (80.0%), race and ethnicity (64.7%), and sex assigned at birth (2499 residents [22.1%]). Overall, 1675 LTCC residents (53.6%) were known to be female, and 369 LTCC residents (11.8%) were confirmed symptomatic during their first infection. Among LTCC residents who were reinfected, the median (IQR) time between the positive test results for their first and second SARS-CoV-2 infections was 7.1 (4.2-8.6) months. For LTCC employees, the median (IQR) age was 41 (30-53) years, 2186 (76.0%) were female, and 1801 (62.6%) were confirmed symptomatic during their first infection (Table 1). Among LTCC employees who were reinfected, the median (IQR) time between the positive tests associated with their first and second SARS-CoV-2 infections was 6.9 (4.8-8.7) months (Table 1). For the general population, the median (IQR) age was 35 (24-52) years, 45 030 (47.6%) were female, and 66 030 (69.9%) were confirmed symptomatic during their first infection. Among members of the general population who were reinfected, median (IQR) time between the positive test results for with their first and second SARS-CoV-2 infections was 7.9 (4.8-10.7) months. Among the general population, 31 895 individuals (33.7%) were residents of high-risk communities and 22 963 individuals (24.3%) were residents of moderate-risk communities, per RIDOH’s zip code–based community COVID-19 risk classification (Table 1).

**Table 1.** Characteristics of Individuals Recovered From COVID-19 and Unvaccinated at the End of the 90-Day Recovery Period

Characteristic	No. (%)		
	General population (n = 94 516)	LTCC employees (n = 2877)	LTCC residents (n = 3124)
Age, median (IQR), y	35 (24-52)	41 (30-53)	81 (71-89)
Sex assigned at birth			
Female	45 030 (47.6)	2186 (76.0)	1675 (53.6)
Male	43 456 (46.0)	395 (13.7)	758 (24.3)
Unknown	6030 (6.4)	296 (10.3)	691 (22.1)
Race and ethnicity			
Asian^a^	1879 (2.0)	54 (1.9)	8 (0.3)
Black^a^	5917 (6.3)	776 (27.0)	54 (1.7)
Hispanic or Latino (any race)	27 625 (29.2)	474 (16.5)	74 (2.4)
White^a^	46 158 (48.8)	1245 (43.3)	966 (30.9)
Other or unknown race^a^^,^^b^	12 937 (13.7)	328 (11.4)	2022 (64.7)
Symptom status during first infection^c^			
Asymptomatic	12 223 (12.9)	903 (31.4)	256 (8.2)
Symptomatic	66 030 (69.9)	1801 (62.6)	369 (11.8)
Unknown	16 263 (17.2)	173 (6.0)	2499 (80.0)
Hospitalization status at first infection			
Not hospitalized	90 149 (95.4)	2817 (97.9)	2768 (88.6)
Hospitalized	4367 (4.6)	60 (2.1)	356 (11.4)
Community COVID-19 risk^d^			
High	31 895 (33.7)	1077 (37.4)	777 (24.9)
Moderate	22 963 (24.3)	785 (27.3)	864 (27.7)
Low	39 258 (41.5)	964 (33.5)	1367 (43.8)
Unknown	400 (0.4)	51 (1.8)	116 (3.7)
Vaccination status at end of follow-up			
CPVS	48 436 (51.2)	1908 (66.3)	1831 (58.6)
Partial CPVS	4247 (4.5)	223 (7.8)	243 (7.8)
Unvaccinated	41 833 (44.3)	746 (25.9)	1050 (33.6)
First vaccine type received			
mRNA-1273	16 752 (17.7)	380 (13.2)	131 (4.2)
BNT162b2	31 316 (33.1)	1676 (58.3)	1891 (60.6)
JNJ-78436735	4615 (4.9)	75 (2.6)	52 (1.7)
Unvaccinated	41 833 (44.3)	746 (25.9)	1050 (33.6)
First infection period^e^			
Wild type period	80 189 (84.8)	2771 (96.3)	3103 (99.3)
Alpha period	9445 (10.0)	84 (2.9)	8 (0.3)
Delta period	4882 (5.2)	22 (0.8)	13 (0.4)
Reinfection status at end of follow-up^e^			
Not reinfected	93 113 (98.5)	2603 (90.5)	2744 (87.8)
Reinfected during wild type period	338 (0.4)	197 (6.8)	321 (10.3)
Reinfected during Alpha period	286 (0.3)	18 (0.6)	27 (0.9)
Reinfected during Delta period	779 (0.8)	59 (2.1)	32 (1.0)
Time between infections among reinfected individuals, median (IQR), mo	7.9 (4.8-10.7)	6.9 (4.8-8.7)	7.1 (4.2-8.6)

Abbreviations: CPVS, completion of primary vaccination series; LTCC, long-term congregate care.

^a^ Individual reported non-Hispanic or unknown ethnicity.

^b^ Includes individuals reporting American Indian or Alaskan Native, Native Hawaiian or Other Pacific Islander, other race, and multiple races.

^c^ Summary of symptom information reported to Rhode Island Department of Health as the reason for testing, during case investigation, and/or through symptom self-monitoring.

^d^ Zip code–based community risk classification created by Rhode Island Department of Health based on community characteristics, such as population density, sociodemographic characteristics, and COVID-19 burden to help guide COVID-19 surveillance and response efforts.

^e^ Predominant COVID-19 strains defined as wild-type strain, February 28, 2020, to March 16, 2021; Alpha strain, March 17 to July 3, 2021; and Delta strain, July 4 to December 9, 2021.

**Figure 3.**
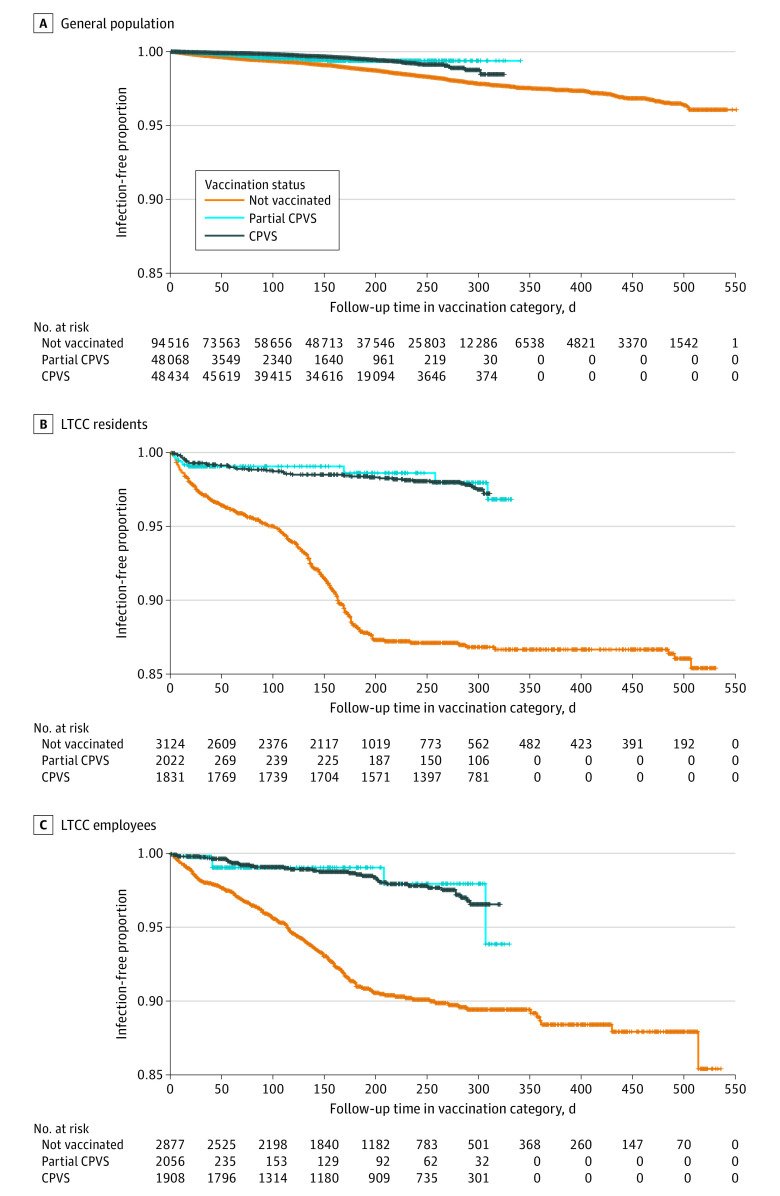
Kaplan-Meier Curves by Vaccination Status CPVS indicates completion of primary vaccination series; LTCC, long-term congregate care.

The cumulative probabilities of reinfection as a function of time since recovery from the primary infection for each subpopulation are shown in eTable 1 in the Supplement. At 9 months after primary infection, the probability of reinfection among those who remained unvaccinated in the general population was 1.9% (95% CI, 1.8%-2.0%). The 9-month probability of reinfection among LTCC residents who remained unvaccinated was 13.0% (95% CI, 12.0%-14.0%) and among LTCC employees who remained unvaccinated was 10.0% (95% CI, 8.8%-11.5%). Overall cumulative reinfection probability by vaccination status appears in Figure 3, which shows substantially lower reinfection probabilities across time. Corresponding incidence rates for cases appear in eTable 2 in the Supplement, which also reports information on hospitalizations and deaths.

The findings of the proportional hazards regression model fit to each subpopulation are presented in eTable 3 in the Supplement, and the interactions of the continuous age and calendar time variables are show in the eFigure in the Supplement. The vaccination status HRs from this model were used to calculate VE estimates (Table 2). VE associated with CPVS (2 doses of mRNA-1273 or BNT162b2 or 1 dose of JNJ-78436735) after recovery from prior SARS-CoV-2 infection was 62% (95% CI, 56%-67%) for the general population , 49% (95% CI, 23% to 66%) for LTCC employees, and 49% (95% CI 26%-65%) for LTCC residents.

**Table 2.** Summary of Reinfections, Hospitalizations, and Deaths, and Vaccine Effectiveness Associated With Protection From COVID-19 Reinfection, by Subpopulation

Vaccination status	Person-time, 100 000 d	Reinfections, hospitalizations, or deaths, No.	Reinfections per 100 000 person-days (95% CI)	Vaccine effectiveness, % (95% CI)^a^
**General population (n = 94 516)**				
Unvaccinated	159.8	1105	6.9 (6.5 to 7.4)	0 [Reference]
Partial CPVS	16.0	55	3.4 (2.6 to 4.5)	52 (37 to 64)
CPVS	83.5	243	2.9 (2.6 to 3.3)	62 (56 to 67)
**LTCC employees (n = 2877)**				
Unvaccinated	5.7	227	40.2 (35.1 to 45.7)	0 [Reference]
Partial CPVS	0.8	11	13.2 (6.6 to 23.7)	55 (11 to 77)
CPVS	3.5	36	10.2 (7.1 to 14.1)	49 (23 to 66)
**LTCC residents (n = 3124)**				
Unvaccinated	6.3	314	50.3 (44.8 to 56.1)	0 [Reference]
Partial CPVS	1.0	25	24.0 (15.5 to 35.3)	33 (−5 to 57)
CPVS	4.9	41	8.4 (6.1 to 11.5)	49 (26 to 65)

Abbreviations: CPVS, completion of primary vaccination series; LTCC, long-term congregate care.

^a^ Calculated by adjusting for age, sex assigned at birth, race and ethnicity, previous hospitalization during the first infection, symptom status during the first infection, zip code–level community risk tier and time of entry into the risk cohort for reinfection relative to the beginning of the pandemic in Rhode Island.

The proportional hazards model was also used to identify factors associated with risk of reinfection (eTable 3 and eFigure in the Supplement). In the general population, individuals who had been hospitalized as a result of their primary SARS-CoV-2 infection had almost 2-fold higher risk of reinfection (AHR, 1.91; 95% CI, 1.56-2.33). Risk of reinfection was lower among men than women (AHR, 0.74; 95% CI, 0.67-0.83); higher among individuals reporting White race or ethnicity compared with Asian, Black, and Hispanic individuals and individuals of other or unknown race; and lower among individuals who were confirmed symptomatic, compared with asymptomatic, during their primary infection (AHR, 0.58; 95% CI, 0.51-0.66). We did not find evidence of similar associations among LTCC residents or employees.

Details on sensitivity analyses are presented in the eAppendix in the Supplement. To assess sensitivity to our definition of CPVS, we restricted the definition of CPVS to receiving both doses at most 42 days apart (eTable 4 in the Supplement). To assess sensitivity to timing of first infection, we repeated the analysis on a sample restricted to individuals whose primary infection occurred during or after December 2020 (eTable 5 in the Supplement). In both cases, VE was similar. A comparison of VE by vaccine type (mRNA-1273 or BNT162b2 vs JNJ-78436735) did not generate strong evidence that estimated effectiveness differed between CVPS via JNJ-78436735 (48%; 95% CI, 26%-63%) was lower than VE associated with mRNA-1273 or BNT162b2 (64%; 95% CI, 58%-69%) in the general population (difference, 16%; 95% CI, −19% to 51%) (eTable 8 in the Supplement). Additionally, there was a higher case rate among individuals aged 20 to 30 years, likely resulting from surveillance testing at colleges and universities (eFigure in the Supplement).

## Discussion

This cohort study is among the first, to our knowledge, to examine VE among people who recovered from COVID-19 and were unvaccinated at the time of their first infection; that is, we examined the effectiveness associated with vaccination after a primary infection. This analysis provides important information for guideline development, particularly relating to residents of LTCC facilities and employees who work in these settings. Previous infection is now taken into account in the development of US Centers for Disease Control and Prevention guidelines^16^ and has been used as an argument against vaccine requirements.^17^

Using a statewide population cohort, we found that probability of reinfection within 9 months of recovery from primary infection with SARS-CoV-2 was approximately 2% in the unvaccinated general population, but 10% in LTCC employees and 13% among LTCC residents. Using a prospective event-history analysis with vaccine exposure as a time-varying covariate, we found that the VE associated with CPVS after recovery from prior SARS-CoV-2 infection was 62% for the general population and 49% for both residents and employees of LTCC facilities. These estimates adjusted for age, sex, race and ethnicity, community COVID-19 risk, symptom and hospitalization status during the primary SARS-CoV-2 infection, and timing of entry into the risk cohort relative to the onset of the pandemic (calendar time). The findings of our statewide cohort study suggest that vaccination after recovery from prior COVID-19 was associated with substantial benefit for preventing subsequent reinfection, with risk reduced by nearly half for all subpopulations. Among LTCC residents and employees, for whom the probability of reinfection at 9 months after primary infection among those who remained unvaccinated was at least 5-fold greater than in the general population (10%-13% vs 2%), this is a particularly notable finding.

Our findings are consistent with a case-control study by Cavenaugh et al^8^ using surveillance data from Kentucky, which found that being unvaccinated was associated with approximately 2-fold higher odds of reinfection compared with being fully vaccinated. A cohort study by Hammerman et al^9^ using electronic medical record data from Israel estimated VE among people who had recovered from prior COVID-19 to be higher (approximately 60% for those aged ≥65 years and 82% for those aged 16-64 years). Another study of health care workers from the United Kingdom found that infection-acquired immunity waned after a year but was consistently higher than 90% for individuals who were subsequently vaccinated.^10^ Given that most hospitalizations and fatalities in our cohort were associated with the first infections, we were not able to conduct formal statistical inference about VE associated with preventing more severe reinfections; however, the observed incidence rates of reinfection-associated COVID-19 hospitalization and death were lower among individuals who were vaccinated compared with individuals who were unvaccinated.

Our findings highlight the continued importance of vaccination, including after recovery from prior COVID-19, among LTCC residents and employees, who are likely at increased risk of reinfection owing to their older age and/or greater exposure to the virus in LTCC settings. Employees and some residents or clients of other settings with greater COVID-19 exposure (eg, health care or correctional environments) may similarly experience high risk of reinfection and benefit from vaccination after recovery from COVID-19.

Our study has several key strengths. The database contains statewide information on positive PCR test results, most positive antigen test results, and nearly all vaccine doses administered in Rhode Island during the study period. The database also has information about whether the individual with positive test results is a resident or employee of a LTCC, a critical subpopulation with respect to SARS-COV-2 infection risk. A second major strength is the prospective nature of the analysis: we were able to follow individuals prospectively in time and calculate VE in terms of relative incidence (1 − *AHR*).

### Limitations

Our analysis has some limitations. First, our analysis was based on diagnosed reinfections; consequently, we are likely underestimating infection rates, particularly in the general population. Our adjustment for age partially accounts for differential testing patterns induced by programs, such as surveillance testing (eg, a higher case rate among individuals aged 20-30 years, likely resulting from surveillance testing at colleges and universities). We may have incomplete information on reinfections identified through home-based antigen testing, but this is expected to be minimal because home-based testing was uncommon until the final weeks of our analysis period. Additionally, our VE estimates could be biased to the extent that testing propensity differs between individuals who are vaccinated and those who are not; for example, individuals who have been previously vaccinated may be less likely to seek testing for a mild infection, leading to an overestimate of VE. Another limitation is that RIDOH’s immunization database does not include most vaccination doses administered by federal entities or out-of-state, which may lead to some misclassification of vaccination status. However, we expect this bias to be conservative, as erroneously classifying some individuals who had been vaccinated as unvaccinated should attenuate our VE estimates. Additionally, children ages 5 to 11 years were not included in our study because they were not eligible for vaccination until the final month of the analysis period. This is an important subgroup for future research. Furthermore, a substantial percentage of LTCC residents were missing data on race and ethnicity (65%) and symptom status during the first infection (80%), owing to heterogeneity in the level of detail reported by each facility, which limited our ability to account for potential confounding by these characteristics.

## Conclusions

The findings of this cohort study suggest that risk of reinfection after recovery from COVID-19 was relatively high among individuals who remained unvaccinated, including the general population and LTCC residents and employees. Vaccination after recovery from COVID-19 was associated with substantial benefit, reducing risk of reinfection by approximately half. Individuals who have recovered from COVID-19 and remain unvaccinated should be encouraged to complete vaccinations, as they are eligible, to reduce their risk of reinfection.

## Notes

Supplement. **eTable 1.** Cumulative Probability of COVID-19 Reinfection, by Subpopulation and Vaccination Status **eTable 2.** Incidence Rate of COVID-19 Reinfection and Reinfection-Associated Hospitalization and Death, After Recovery From Prior COVID-19, by Subpopulation and Vaccination Status **eTable 3.** Hazard of Reinfection From Proportional Hazard Regression Models Fit to Data From Each Subpopulation **eFigure.** Partial Association Plots for Age and Cohort Entry Time Interactions Estimated Using Spline Terms in Cox Regression, by Subpopulation **eAppendix.** Description of Sensitivity Analyses **eTable 4.** Sensitivity Analysis: Time Between Vaccine Doses in a 2-Dose Series Limited to a Maximum of 42 Days **eTable 5.** Sensitivity Analysis: Restrict Analysis Individuals Who Entered the Risk Cohort After Vaccine Rollout Only **eTable 6.** Characteristics of Patients Removed From First Sensitivity Analysis that Excludes Individuals With More Than 42 Days Between First and Second Dose of Either mRNA Vaccine **eTable 7.** Characteristics of Individuals Removed From Second Sensitivity Analysis Excluding Those Whose First Infection Occurred Prior to Vaccine Rollout **eTable 8.** Vaccine Effectiveness Associated With Protection Against COVID-19 Reinfection by Subpopulation, Stratified by Vaccine Type, Estimated Using a Cox Proportional Hazards Regression
