# Supplementary material for: Effectiveness Associated With Vaccination After COVID-19 Recovery in Preventing Reinfection
Source: JAMA Netw Open. 2022 Jul 27;5(7):e2223917. doi: 10.1001/jamanetworkopen.2022.23917 (PMC9331088; doi:10.1001/jamanetworkopen.2022.23917)
Supplement: Supplement. — eTable 1. Cumulative Probability of COVID-19 Reinfection, by Subpopulation and Vaccination Status eTable 2. Incidence Rate of COVID-19 Reinfection and Reinfection-Associated Hospitalization and Death, After Recovery From Prior COVID-19, by Subpopulation and Vaccination Status eTable 3. Hazard of Reinfection From Proportional Hazard Regression Models Fit to Data From Each Subpopulation eFigure. Partial Association Plots for Age and Cohort Entry Time Interactions Estimated Using Spline Terms in Cox Regression, by Subpopulation eAppendix. Description of Sensitivity Analyses eTable 4. Sensitivity Analysis: Time Between Vaccine Doses in a 2-Dose Series Limited to a Maximum of 42 Days eTable 5. Sensitivity Analysis: Restrict Analysis Individuals Who Entered the Risk Cohort After Vaccine Rollout Only eTable 6. Characteristics of Patients Removed From First Sensitivity Analysis that Excludes Individuals With More Than 42 Days Between First and Second Dose of Either mRNA Vaccine eTable 7. Characteristics of Individuals Removed From Second Sensitivity Analysis Excluding Those Whose First Infection Occurred Prior to Vaccine Rollout eTable 8. Vaccine Effectiveness Associated With Protection Against COVID-19 Reinfection by Subpopulation, Stratified by Vaccine Type, Estimated Using a Cox Proportional Hazards Regression [file jamanetwopen-e2223917-s001.pdf]

Supplemental Online Content

Lewis N, Chambers LC, Chu HT, et al. Effectiveness associated with vaccination after COVID-19 recovery in preventing reinfection. *JAMA Netw Open*. 2022;5(7):e2223917. doi:10.1001/jamanetworkopen.2022.23917

eTable 1. Cumulative Probability of COVID-19 Reinfection, by Subpopulation and Vaccination Status

eTable 2. Incidence Rate of COVID-19 Reinfection and Reinfection-Associated Hospitalization and Death, After Recovery From Prior COVID-19, by Subpopulation and Vaccination Status

eTable 3. Hazard of Reinfection From Proportional Hazard Regression Models Fit to Data From Each Subpopulation

eFigure. Partial Association Plots for Age and Cohort Entry Time Interactions Estimated Using Spline Terms in Cox Regression, by Subpopulation

eAppendix. Description of Sensitivity Analyses

eTable 4. Sensitivity Analysis: Time Between Vaccine Doses in a 2-Dose Series Limited to a Maximum of 42 Days

eTable 5. Sensitivity Analysis: Restrict Analysis Individuals Who Entered the Risk Cohort After Vaccine Rollout Only

eTable 6. Characteristics of Patients Removed From First Sensitivity Analysis that Excludes Individuals With More Than 42 Days Between First and Second Dose of Either mRNA Vaccine

eTable 7. Characteristics of Individuals Removed From Second Sensitivity Analysis Excluding Those Whose First Infection Occurred Prior to Vaccine Rollout

eTable 8. Vaccine Effectiveness Associated With Protection Against COVID-19 Reinfection by Subpopulation, Stratified by Vaccine Type, Estimated Using a Cox Proportional Hazards Regression

This supplemental material has been provided by the authors to give readers additional information about their work.

© 2022 Lewis N et al. *JAMA Network Open.*

eTable 1.
Cumulative Probability of COVID-19 Reinfection, by Subpopulation and Vaccination Status

	3 Months	6 Months	9 Months
General population (N = 94,516)			
Unvaccinated	0.6% (0.5%, 0.6%)	1.2% (1.1%, 1.3%)	1.9% (1.8%, 2.0%)
CPVS	0.2% (0.1%, 0.2%)	0.5% (0.4%, 0.6%)	1.0% (0.8%, 1.1%)
LTCC employees (N=2,877)			
Unvaccinated	4.0% (3.3%, 4.8%)	8.8% (7.6%, 9.9%)	10.0% (8.8%, 11.5%)
CPVS	0.9% (0.5%, 1.4%)	1.3% (0.8%, 1.9%)	2.5% (1.6%, 3.4%)
LTCC residents (N = 3,124)			
Unvaccinated	4.8% (4.0%, 5.6%)	12.0% (10.0%, 13.0%)	13.0% (12.0%, 14.0%)
CPVS	1.2% (0.7%, 1.7%)	1.6% (1.0%, 2.2%)	2.0% (1.3%, 2.7%)

Abbreviations: CI, confidence interval; COVID-19, Coronavirus Disease 2019; LTCC, long-term congregate care. CPVS, Completed Primary Vaccination Series.

© 2022 Lewis N et al. *JAMA Network Open.*

eTable 2.

Incidence Rate of COVID-19 Reinfection and Reinfection-Associated Hospitalization and Death, After Recovery From Prior COVID-19, by Subpopulation and Vaccination Status

	Person time (100,000 Days)	Sample size, number of reinfections / hospitalizations / deaths[b]	Reinfections per 100,000 person days (95% CI)[a]	Hospitalizations per 100,000 person days (95% CI)[a]	Deaths per 100,000 person days (95% CI)[a]
General population		N = 94,516			
Unvaccinated	159.8	1,105 / 76 / 5	6.9 (6.5, 7.4)	0.5 (0.1, 0.4)	0.03 (0.01, 0.07)
Partial CPVS	16.0	55 / 5 / 0	3.4 (2.6, 4.5)	0.3 (0.1, 0.7)	0
CPVS	83.5	243 / 19 / <5	2.9 (2.6, 3.3)	0.2 (0.1, 0.4)	N/A[b]
LTCC employees		N = 2,877			
Unvaccinated	5.7	227 / 7 / 0	40.2 (35.1, 45.7)	1.2 (0.5, 2.6)	0
Partial CPVS	0.8	11 / 0 / 0	13.2 (6.6, 23.7)	0	0
CPVS	3.5	36 / 0 / 0	10.2 (7.1, 14.1)	0	0
LTCC residents		N = 3,124			
Unvaccinated	6.3	314 / 36 / 12	50.3 (44.8, 56.1)	5.8 (4.0, 8.0)	1.9 (1.0, 3.4)
Partial CPVS	1.0	25 / <5 / 0	24.0 (15.5, 35.3)	N/A[b]	0
CPVS	4.9	41 / 5 / <5	8.4 (6.1, 11.5)	1.0 (0.3, 2.4)	N/A[b]

Abbreviations: CI, confidence interval; COVID-19, Coronavirus Disease 2019; LTCC, long-term congregate care. CPVS, Completed Primary Vaccination Series; N/A, not available.

[a] 95% confidence intervals were calculated using a Poisson test.

[b] Counts between 1 and 4, and calculations based on those counts, are suppressed in accordance with RIDOH's Small Numbers Policy.

© 2022 Lewis N et al. *JAMA Network Open.*

eTable 3.

Hazard of Reinfection From Proportional Hazard Regression Models Fit to Data From Each Subpopulation

Covariate	General population N=94,516 (95% CI)	LTCC employees N=2,877 (95% CI)	LTCC residents N=3,124 (95% CI)
Vaccination status			
Unvaccinated	Ref.	Ref.	Ref.
Partial CPVS	0.48 (0.37, 0.64)	0.45 (0.23, 0.89)	0.67 (0.43, 1.05)
CPVS	0.38 (0.33, 0.44)	0.51 (0.34, 0.77)	0.51 (0.35, 0.74)
Sex			
Female	Ref.	Ref.	Ref.
Male	0.74 (0.67, 0.83)	1.17 (0.85, 1.62)	0.85 (0.65, 1.13)
Unknown	0.20 (0.13, 0.29)	0.11 (0.05, 0.23)	0.76 (0.55, 1.05)
Race/ethnicity			
White[a]	Ref.	Ref.	Ref.
Hispanic or Latino (any race)	0.81 (0.70, 0.95)	1.06 (0.74, 1.53)	0.89 (0.47, 1.70)
Asiana	0.47 (0.28, 0.79)	0.71 (0.29, 1.77)	1.64 (0.40, 6.64)
Black or African Americana	0.92 (0.74, 1.15)	0.88 (0.62, 1.25)	0.90 (0.45, 1.80)
Other or unknown race[a]	0.57 (0.46, 0.70)	0.51 (0.30, 0.86)	0.66 (0.52, 0.83)
Community COVID-19 risk[b]			
High	Ref.	Ref.	Ref.
Moderate	1.13 (0.97, 1.32)	1.08 (0.80, 1.47)	1.18 (0.89, 1.55)
Low	1.11 (0.95, 1.30)	1.00 (0.71, 1.42)	1.16 (0.89, 1.52)
Unknown	0.29 (0.07, 1.17)	0.76 (0.27, 2.12)	0.43 (0.19, 0.98)
Symptom status during first infection[c]			
Asymptomatic	Ref.	Ref.	Ref.
Symptomatic	0.58 (0.51, 0.66)	0.76 (0.58, 0.99)	1.28 (0.81, 2.02)
Unknown	0.77 (0.65, 0.93)	1.47 (0.98, 2.21)	1.12 (0.75, 1.67)
Hospitalization during first infection			
Not hospitalized	Ref.	Ref.	Ref.
Hospitalized	1.91 (1.56, 2.33)	0.99 (0.44, 2.22)	0.91 (0.65, 1.28)

Abbreviations: CI, Confidence Interval; COVID-19, Coronavirus Disease 2019; RIDOH, Rhode Island Department of Health; CPVS, Completion of Primary Vaccination Series.

[a] Patient reported non-Hispanic or unknown ethnicity.

[b] Summary of symptom information reported to RIDOH as the reason for testing, during case investigation, and/or through symptom self-monitoring.

[c] ZIP-code-based community risk classification created by RIDOH based on community characteristics such as population density, sociodemographics, and COVID-19 burden to help guide COVID-19 surveillance and response efforts.

© 2022 Lewis N et al. *JAMA Network Open.*

eFigure.
Partial Association Plots for Age and Cohort Entry Time Interactions Estimated Using Spline Terms in Cox Regression, by Subpopulation

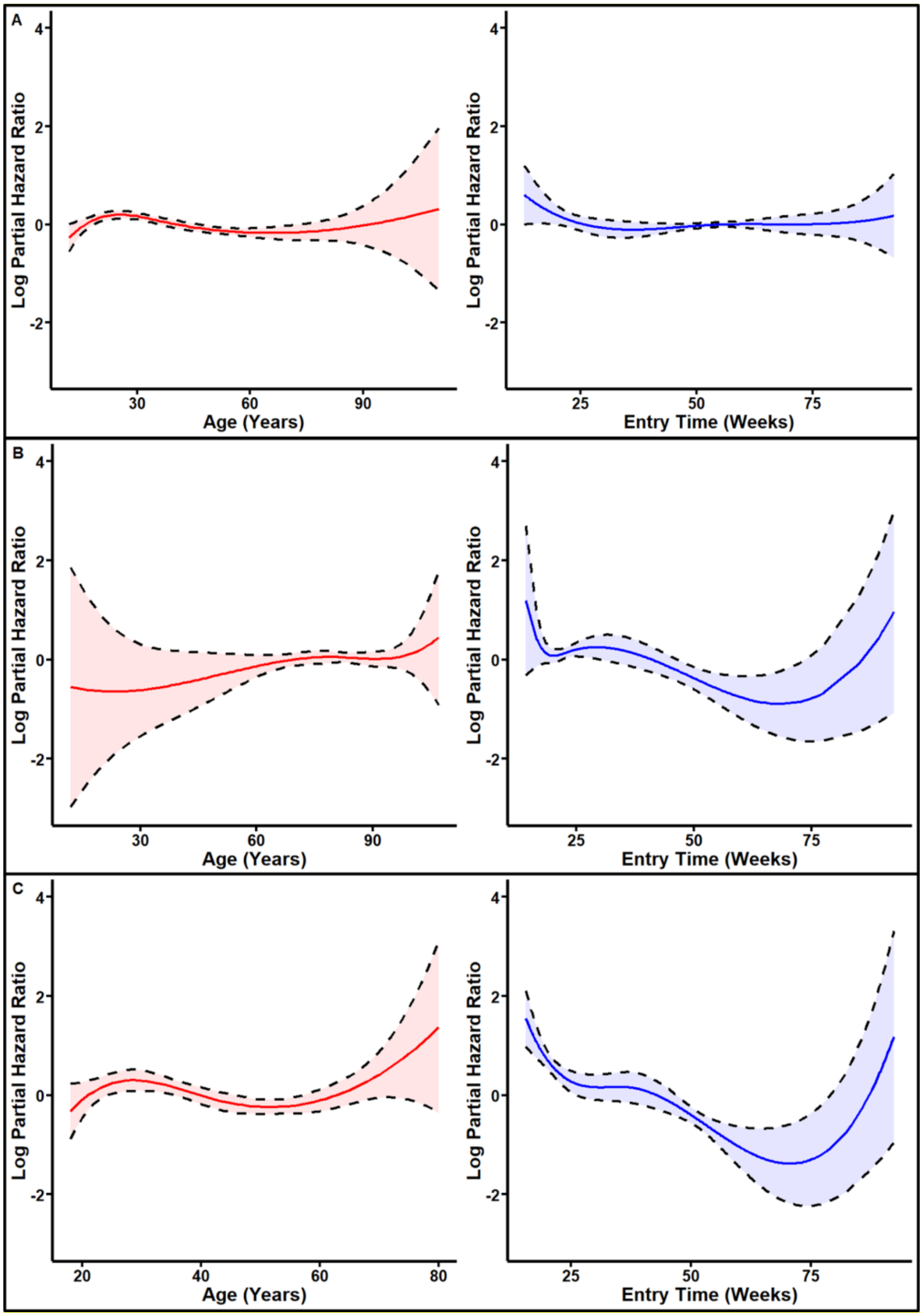

Abbreviations: LTCC, long-term congregate care; CPVS, Completion of Primary Vaccination Series.

© 2022 Lewis N et al. *JAMA Network Open.*

eAppendix. Description of Sensitivity Analyses

We conducted two sensitivity analyses. The first assesses sensitivity to our definition of completion of primary vaccination series for vaccines requiring two doses; the second examines the impact of including data during a period where vaccines were not available.

The first sensitivity analysis is motivated by the idea that, for the two-dose regimes, a person whose second dose is administered after a long delay has potentially lost a significant amount of vaccine-conferred immunity. This could result in downward bias of the vaccine effect. Therefore, we removed any people who completed the primary vaccination series but received their second dose at least 42 days after their first dose. This resulted in the removal of 1,273 people. After pruning the data, the analysis followed the same schematic as described in the paper. The results are shown in eTable 4 and are consistent with the results of the primary analysis (Table 2 in the manuscript).

The second sensitivity analysis addresses the possibility that the estimated effect of vaccination might be affected because vaccines were not available prior to December 2020 (i.e. during that time, there were no individuals at risk for infection who also were vaccinated).   If the background risk of infection is systematically different during that period, the vaccine effect could be biased. For example, if background infection risk is higher prior to December 2020, our reported vaccine effect could be biased upward. To address this, we restricted the observation period to run from December 14, 2020, to December 9, 2021. This resulted in the removal of 404 people. The analysis of this subset is reported in eTable 5 and are consistent with the results of the primary analysis reported in Table 2 in the manuscript.

eTables 6 and 7 summarize the characteristics of those removed from main sample to construct the datasets used in the sensitivity analyses.

© 2022 Lewis N et al. *JAMA Network Open.*

eTable 4.
Sensitivity Analysis: Time Between Vaccine Doses in a 2-Dose Series Limited to a Maximum of 42 Days

	**Vaccine effectiveness (95% CI)**
General population	
Partial CPVS	56% (41%, 68%)
CPVS	62% (56%, 67%)
LTCC employees	
Partial CPVS	51% (3%, 75%)
CPVS	47% (19%, 65%)
LTCC residents	
Partial CPVS	39% (0%, 63%)
CPVS	47% (23%, 64%)

Abbreviations: CI, confidence interval; COVID-19, Coronavirus Disease 2019; CPVS, competed primary vaccination series; LTCC, long-term congregate care.

© 2022 Lewis N et al. *JAMA Network Open.*

eTable 5.

Sensitivity Analysis: Restrict Analysis Individuals Who Entered the Risk Cohort After Vaccine Rollout Only

	**Vaccine effectiveness (95% CI)**
General population	
Partial CPVS	49% (33%, 61%)
CPVS	64% (58%, 69%)
LTCC employees	
Partial CPVS	50% (0%, 75%)
CPVS	49% (23%, 67%)
LTCC residents	
Partial CPVS	18% (-29%, 48%)
CPVS	45% (19%, 63%)

Abbreviations: CI, confidence interval; COVID-19, Coronavirus Disease 2019; CPVS, competed primary vaccination series; LTCC, long-term congregate care.

© 2022 Lewis N et al. *JAMA Network Open.*

eTable 6.

Characteristics of Patients Removed From First Sensitivity Analysis that Excludes Individuals With More Than 42 Days Between First and Second Dose of Either mRNA Vaccine

**Characteristic**	**General population** N=1,104 n (%)	**LTCC employees** N=86 n (%)	**LTCC residents** N=83 n (%)
Age (years), median (IQR)	35 (23-51)	42 (32-52)	77 (68-86)
Sex assigned at birth			
Female	544 (49.3)	70 (81.4)	46 (55.4)
Male	487 (44.1)	9 (10.5)	19 (22.9)
Unknown	73 (6.6)	7 (8.1)	18 (21.7)
Race/ethnicity			
Hispanic or Latino (any race)	477 (43.2)	32 (37.2)	27 (32.5)
Black or African American[a]	387 (35.1)	18 (20.9)	2 (2.4)
White[a]	21 (1.9)	1 (1.2)	0 (0.0)
Asian[a]	74 (6.7)	23 (26.7)	3 (3.6)
Other or unknown race[a]	145 (13.1)	12 (14.0)	51 (61.4)
Symptom status during first infection[b]			
Asymptomatic	131 (11.9)	27 (31.4)	2 (2.4)
Symptomatic	810 (73.4)	55 (64.0)	17 (20.5)
Unknown	163 (14.8)	4 (4.7)	64 (77.1)
Community COVID-19 risk[c]			
High	453 (41.0)	31 (36.0)	21 (25.3)
Moderate	222 (20.1)	28 (32.6)	21 (25.3)
Low	427 (38.7)	27 (31.4)	37 (44.6)
Unknown	2 (0.2)	0 (0.0)	4 (4.8)
Vaccination status at end of follow-up			
CPVS	1,079 (97.7)	75 (87.2)	65 (78.3)
Partial CPVS	8 (0.7)	0 (0.0)	4 (4.8)
Unvaccinated	17 (1.5)	11 (12.8)	14 (16.9)
First vaccine type received			
Moderna	491 (44.5)	16 (18.6)	12 (14.5)
Pfizer	596 (54.0)	59 (68.6)	57 (68.7)

© 2022 Lewis N et al. *JAMA Network Open.*

Janssen	0 (0.0)	0 (0.0)	0 (0.0)
Unvaccinated	17 (1.5)	11 (12.8)	14 (16.9)
Hospitalization status at first infection			
Not hospitalized	1,030 (93.3)	81 (94.2)	73 (88.0)
Hospitalized	74 (6.7)	5 (5.8)	10 (12.0)
First infection period [d]			
Infected during wild type period	1,042 (94.4)	85 (98.8)	83 (100.0)
Infected during Alpha period	62 (5.6)	1 (1.2)	0 (0.0)
Infected during Delta period	0 (0.0)	0 (0.0)	0 (0.0)
Reinfection status at end of follow-up[d]			
Not reinfected	1,077 (97.6)	75 (87.2)	65 (78.3)
Reinfected during wild type period	8 (0.7)	10 (11.6)	15 (18.1)
Reinfected during Alpha period	11 (1.0)	1 (1.2)	2 (2.4)
Reinfected during Delta period	8 (0.7)	0 (0.0)	1 (1.2)
Months between infections among reinfected individuals, median (IQR)	7.9 (4.8-10.7)	6.9 (4.8-8.7)	7.1 (4.2-8.6)

Abbreviations: COVID-19, Coronavirus Disease 2019; RIDOH, Rhode Island Department of Health; IQR, Interquartile Range; CPVS, Completion of Primary Vaccination Series.
[a] Patient reported non-Hispanic or unknown ethnicity.
[b] Summary of symptom information reported to RIDOH as the reason for testing, during case investigation, and/or through symptom self-monitoring.
[c] ZIP-code-based community risk classification created by RIDOH based on community characteristics such as population density, sociodemographics, and COVID-19 burden to help guide COVID-19 surveillance and response efforts.
[d] Predominant COVID-19 strains defined as follows: wild type strain - 2/28/2020-03/16/2021, Alpha strain - 03/17/2021-07/03/2021, and Delta strain – 07/04/2021-12/9/2021.

© 2022 Lewis N et al. *JAMA Network Open.*

eTable 7.
Characteristics of Individuals Removed From Second Sensitivity Analysis Excluding Those Whose First Infection Occurred Prior to Vaccine Rollout

**Characteristic**	**General population** N=145 n (%)	**LTCC employees** N=106 n (%)	**LTCC residents** N=153 n (%)
Age (years), median (IQR)	42 (28-59)	44 (32-57)	81 (73-90)
Sex assigned at birth			
Female	81 (55.9)	82 (77.4)	79 (51.6)
Male	57 (39.3)	19 (17.9)	33 (21.6)
Unknown	7 (4.8)	5 (4.7)	41 (26.8)
Race/ethnicity			
Hispanic or Latino (any race)	44 (30.3)	48 (45.3)	65 (42.5)
Black or African American[a]	71 (49.0)	20 (18.9)	3 (2.0)
White[a]	3 (2.1)	1 (0.9)	0 (0.0)
Asian[a]	17 (11.7)	31 (29.2)	1 (0.7)
Other or unknown race[a]	10 (6.9)	6 (5.7)	84 (54.9)
Symptom status during first infection[b]			
Asymptomatic	39 (26.9)	40 (37.7)	8 (5.2)
Symptomatic	81 (55.9)	55 (51.9)	38 (24.8)
Unknown	25 (17.2)	11 (10.4)	107 (69.9)
Community COVID-19 risk[c]			
High	82 (56.6)	51 (48.1)	36 (23.5)
Moderate	28 (19.3)	26 (24.5)	54 (35.3)
Low	35 (24.1)	27 (25.5)	61 (39.9)
Unknown	0 (0.0)	2 (1.9)	2 (1.3)
Hospitalization status at first infection			
Not hospitalized	121 (83.4)	101 (95.3)	138 (90.2)
Hospitalized	24 (16.6)	5 (4.7)	15 (9.8)
Months between infections among reinfected individuals, median (IQR)	4.7 (3.6-6.2)	5.3 (3.7-6.8)	4.9 (3.6-6.6)

Abbreviations: COVID-19, Coronavirus Disease 2019; RIDOH, Rhode Island Department of Health; IQR, Interquartile Range; CPVS, Completion of Primary Vaccination Series.
[a] Patient reported non-Hispanic or unknown ethnicity.
[b] Summary of symptom information reported to RIDOH as the reason for testing, during case investigation, and/or through symptom self-monitoring.
[c] ZIP-code-based community risk classification created by RIDOH based on community characteristics such as population density, sociodemographics, and COVID-19 burden to help guide COVID-19 surveillance and response efforts.
Note: for Table S2 we exclude information about first strain infection and vaccination status at reinfection. This is because 1) we exclude anybody before Dec 14 2020 so obviously there first strain was the wild type. Secondly, given they were excluded before vaccine rollout there vax status is unvaccinated and second infection strain is wild as well.

© 2022 Lewis N et al. *JAMA Network Open.*

eTable 8.

Vaccine Effectiveness Associated With Protection Against COVID-19 Reinfection by Subpopulation, Stratified by Vaccine Type, Estimated Using a Cox Proportional Hazards Regression

	**Vaccine effectiveness (95% CI)**
**General population**	
Partial CPVS, Moderna/Pfizer	52% (37%, 64%)
CPVS, Moderna/Pfizer	64% (58%, 69%)
CPVS, Janssen	48% (26%, 63%)
**LTCC employees**	
Partial CPVS, Moderna/Pfizer	33% (-5%, 57%)
CPVS, Moderna/Pfizer	49% (26%, 65%)
CPVS, Janssen	57% (-211%, 94%)
**LTCC residents**	
Partial CPVS, Moderna/Pfizer	55% (11%, 77%)
CPVS, Moderna/Pfizer	52% (26%, 68%)
CPVS, Janssen	-68% (-415%, 45%)

Abbreviations: CI, confidence interval; COVID-19, Coronavirus Disease 2019; LTCC, long-term congregate care; CPVS, Completion of Primary Vaccination Series.

© 2022 Lewis N et al. *JAMA Network Open.*
